# Frequency-Dependent Changes of the Resting BOLD Signals Predicts Cognitive Deficits in Asymptomatic Carotid Artery Stenosis

**DOI:** 10.3389/fnins.2018.00416

**Published:** 2018-06-21

**Authors:** Feng Xiao, Tao Wang, Lei Gao, Jian Fang, Zhenmeng Sun, Haibo Xu, Junjian Zhang

**Affiliations:** ^1^Department of Radiology, Zhongnan Hospital of Wuhan University, Wuhan, China; ^2^Department of Neurology, Zhongnan Hospital of Wuhan University, Wuhan, China; ^3^Department of Neurology, The First College of Clinical Medical Science, China Three Gorges University, Yichang, China

**Keywords:** asymptomatic carotid artery stenosis, cognitive, frequency, resting BOLD, diagnosis model, prediction model

## Abstract

“Asymptomatic” carotid artery stenosis (aCAS) patients usually have cognitive impairment in the domains of executive, psychomotor speed, and memory function. However, the pathophysiology of this impairment in aCAS patients is still unclear. In this study, amplitude of low-frequency fluctuation (ALFF) method was used based on resting-state blood oxygenation level dependent (BOLD) signals, to investigate local brain activity in 19 aCAS patients and 24 healthy controls, aimed to explore this pathophysiology mechanism. We analyzed this intrinsic activity in four individual frequency bands: Slow-2 (0.198–0.25 Hz), Slow-3 (0.073–0.198 Hz), Slow-4 (0.027–0.073 Hz), and Slow-5 (0.01–0.027 Hz). The aCAS-related ALFF changes were mainly distributed in (1) cortical midline structure, including bilateral dorsomedial prefrontal (dmPFC), cingulate cortex (CC) and precuneus (PCu); (2) hippocampus and its adjacent structures, including bilateral hippocampus, thalamus and medial temporal regions. We found these spatial patterns were frequency-dependent. Significant interaction between frequency and group was found distributed in left putamen, triangle part of inferior temporal and bilateral precentral/postcentral gyrus when Slow-4 and Slow-5 were considered. The delay recall ability of aCAS patient was significantly positive correlated to the mean ALFF in dmPFC within Slow-4 band and the mean ALFF in the bilateral hippocampus within Slow-3 band, respectively. We also found the Montreal Cognitive Assessme score of aCAS patient was significantly positive correlated to the mean ALFF in right fusiform and parahippocampus within Slow-3 band. Furthermore, we built the automatic diagnosis and prediction models based on support vector machine (SVM) and back propagation neural network (BPNN), respectively. Both two types of models could achieve relatively competent performance, which meant the frequency-dependent changes in ALFF could not only reveal the pathophysiology mechanism of cognitive impairment of aCAS, but also could be used as neuroimaging marker in the analysis of cognition impairment for aCAS patients.

## Introduction

Carotid artery stenosis with no symptoms of transient ischemic attack or stroke is so called “asymptomatic” (Inzitari et al., 2000). However, “asymptomatic” carotid artery stenosis (aCAS) patients usually show cognitive impairment in the executive function, psychomotor speed and memory (Romero et al., 2009; Sztriha et al., 2009; Popovic et al., 2011), which implies aCAS is not asymptomatic in practice. aCAS may be a underlying risk factor for cognitive impairment (Chang et al., 2013; Everts et al., 2014).

Generally, CAS is a chronic process, and the impairment of neuron is long-lasting. During this persistent process, there is also enough time for neuron being compensated and repaired by different patterns, such as collateral circulation and vascular self-regulation (Deweese et al., 1970), which make the process of cognitive impairment complicated. Until now, the pathophysiological mechanism of cognitive impairment is poorly understood.

Blood oxygenation level dependent (BOLD) is related to the neural activity in brain through the detection of the combination between oxygen and hemoglobin in blood. Low frequency BOLD signal can reflect the spontaneous neural activity in brain, which can be used to reveal the relationship between the cognitive impairment and their associated structurally/functionally impaired brain regions.

In recent years, resting-state functional magnetic resonance imaging (RfMRI) method based on BOLD signals has been extensively used in neuropsychiatric researches. Among the RfMRI analysis methods, amplitude of low-frequency fluctuation (ALFF) (Zang et al., 2007) is effective and powerful for examining disease-related neural activity in local brain region and has been successfully used in the study of cognition impairment disease, e.g., schizophrenia (Guo et al., 2015), subcortical ischemic vascular disease (Li et al., 2014), major depressive disorder (Liu et al., 2013), Alzheimer’s disease (He et al., 2007), attention deficit hyperactivity disorder (Zang et al., 2007) and in our previous work (Wang et al., 2016), we reported some initial results by using ALFF method.

Different oscillatory bands usually have different generation mechanism and different physiological functions (Buzsaki and Draguhn, 2004). Therefore, it is meaningful to segment and separate the different frequency bands in low frequency oscillation (LFO) analysis of this work.

In this paper, (1) we use whole-brain, full bandwidth, ALFF-based method to explore the difference of local brain activities between aCAS patients and healthy control, aimed to reveal the pathophysiological mechanism of cognitive impairment in aCAS. To find the temporal and spatial characteristics of the oscillation of neural activities in the brain of aCAS patients, the whole frequency band (0.01–0.25 Hz) of BOLD signals was separated into four individual frequency bands (Penttonen and Buzsáki, 2003; Buzsaki and Draguhn, 2004): Slow-5 (0.01–0.027 Hz), Slow-4 (0.027–0.073 Hz), Slow-3 (0.073–0.198 Hz) and Slow-2 (0.198–0.25 Hz). Then, the spatial distributions of between-group differences were analyzed within each sub-band, respectively. With the comparison of ALFF spatial distribution between aCAS group and control group, we found the ALFF values in specific brain regions correlated to the cognition impairment of aCAS within each sub-frequency band and their correlation to different cognition impairment. (2) we built the automatic diagnosis model and cognition impairment prediction model for the aCAS patients by using support vector machine (SVM) (Cristianini, 2000) and backpropagation neural network (BPNN) (Hagan, 1996), respectively. The mean ALFF values of the brain regions with significant between-group difference were used as neuroimaging markers to classify and predict the cognition level of subjects.

## Materials and Methods

### Participants

All the participants were collected between January 2015 and June 2016. aCAS patients were recruited from Department of Neurology, Zhongnan hospital of Wuhan university. The inclusion criteria include the following: (1) age from 55 to 80 years; (2) ICA stenosis degree ≥ 70%; (3) right hand dominance; (4) being free of stroke, TIA, dementia, or depression; (5) Modified Rankin Scale: score 0 or 1; and (6) no major psychiatric disease or other medical conditions. The exclusion criteria were (1) contralateral internal carotid artery stenosis ≥ 50%; (2) posterior circulation diseases; (3) MMSE < 26; (4) functional disability (Modified Rankin Scale ≥ 2); (5) severe systemic diseases and neuropsychiatric diseases (such as congestive heart failure and history of stroke); (6) any contraindications for MR scan (e.g., metal implants); and (7) low education level (<6 years). Angiography technique was used to determine the presence of stenosis in our subjects, while North American Symptomatic Carotid Endarterectomy (NASCET) was used to evaluate the degree of stenosis in the patients. Demographics-matched healthy controls were enrolled from the nearby residents by the advertisement surrounding Wuhan University.

Before data acquisition, Routine morphological MRI examination was used on all subjects. For the patients group, we exclude the subjects with new cerebral infarction and/or old infarct lesion diameter > 1.5 cm; while for the control group, we exclude the subjects with ischemic or hemorrhagic stroke, lacunar infarcts, and white matter lesions. Finally, 19 aCAS patients and 24 controls were included in the two group of our data set. The detailed demographics of the two groups were shown in **Table 1**.

**Table 1:** Demographics and cognitive test scores for the subjects in this study.

Characteristics	Patients (*n* = 19)	Control (*n* = 24)	*P*-value
Age (years)	68 ± 5.6	64.5 ± 7.3	0.08
Male: female	15:4	19:5	>0.99
Education (years)	9.9 ± 3.3	10.9 ± 3.4	0.21
Hypertension	19	18	0.70
Diabetes mellitus	4	4	>0.99
Hypercholesterolemia	13	12	0.64
Stenotic side			
Left	7	N/A	
Right	12	N/A	
MMSE	26.8 ± 0.7	27.4 ± 0.7	0.02
MoCA	23.3 ± 1.2	24.2 ± 1.6	0.02
Digit span test (DST)			
Forward digit span (FDS)	5.8 ± 1.0	6.5 ± 0.9	0.04
Backward digit span (BDS)	3.8 ± 0.8	4.5 ± 0.8	0.02
Rey auditory verbal learning test (RAVLT)			
Immediate recall (IR)	31.0 ± 4.5	35.8 ± 5.6	<0.01
Delayed recall (DR)	4.6 ± 1.6	6.5 ± 1.1	<0.01
digital symbol substitution test (DSST)	28.0 ± 4.7	31.5 ± 5.5	0.03


This study was approved by the local Medical Ethics Committee in Zhongnan Hospital of Wuhan University, and informed written consent was signed by all participants.

### Cognition Assessment

Cognition assessments were performed using neuropsychological scales within 7 days after MRI scan. Seven neuropsychological scales were used in this study.

(1) MMSE (Pangman et al., 2000) and MoCA Beijing Version (Yu et al., 2012) were utilized to assess the global cognition. (2) Digit Span Test (DST) (Firat, 2010) is used to measure working memory’s number storage capacity.

In this test, all the subjects were asked to recall a series of oral numbers with different digits. In forward digit span (FDS), subjects were required to retell the numbers in forward order. In the backward digit span (BDS), subjects were required to retell the numbers in backward order.

(3) Rey Auditory Verbal Learning Test (RAVLT) (Vakil et al., 2004) was applied to evaluate the ability of verbal learning and memory.

In this test, the subjects should try to repeat the words as much as he/she can remember. This procedure was repeated five times and then followed a delayed recall after 30 min. The sum number of the correct words Immediately Recall (IR) and Delayed Recall (DR) during the first five repeats were recorded, respectively.

(4) Digital Symbol Substitution Test (DSST) (Lezak, 2004) was utilized to test feel/movement speed, sustained attention and short-term memory.

In this test, subjects were required to convert numbers into symbols in a given time and the correct conversions completed in 90 s were recorded.

The detailed values of neuropsychological scales in the two groups were shown in **Table 1**.

### Data Acquisition

RfMRI data were collected using Siemens 3.0-T MR scanner (MAG-NETOM Trio Tim System) in Department of Radiology, Zhongnan Hospital of Wuhan University. The resting-state functional images were acquired using a EPI-BOLD sequence (repetition time: 2000 ms; echo time: 30 ms; slice thickness: 3.8 mm; gap: 1 mm; number of slices: 33; field of view: 240 mm × 240 mm; data matrix: 64 × 64; flip angle: 90°). All participants were asked to lie relaxed in the scanner, close their eyes but stay awake, try not to think of anything. During the scanning, we found nobody fall asleep or being uncomfortable.

### Data Preprocessing

RfMRI data preprocessing was completed using Data Processing Assistant for resting-state fMRI (DPABI 2.3^1^). In this study, the preprocessing procedure included eight steps: (1) the removal of first ten volumes; (2) slice timing; (3) head-motion correction; (4) spatial normalization to the Montreal Neurological Institute (MNI) space; (5) re-sampling to 3 mm × 3 mm × 3 mm; (6) spatial smoothing with a 6 mm Gaussian kernel; (7) linear detrending; (8) nuisance regression. Each of the step is one of the functional modules in DPABI software.

Subjects with a maximum angular rotation of more than 1° or a maximum displacement of more than 1 mm in *x, y*, or *z* axis for any of the remain 230 slices were excluded from this study. No subject was excluded according to this criterion.

### ALFF Calculation

REST 1.8 (Yan et al., 2016) was used to calculate the ALFF value. To explore the frequency dependent characteristics of the RfMRI signals, in this study we divided the whole frequency range (0.01–0.25 Hz) into four individual frequency band (Wei et al., 2014): Slow-5 (0.01–0.027 Hz), Slow-4 (0.027–0.073 Hz), Slow-3 (0.073–0.198 Hz), and Slow-2 (0.198–0.25 Hz). For each voxel in each slice of each subject, the ALFF value in the whole frequency range and four sub-bands was calculated separately.

### Statistical Analysis

IBM SPSS 20.0 and SPM8 were used to perform Demographically statistical analyses. Continuous variables were assessed with Mann–Whitney test or two-sample *t*-test. Categorical variables were assessed with Chi-squared or Fisher exact test if the expected number was ≤5. Significance was defined as *P* < 0.05. Education and age were defined as covariates in all tests involving cognition.

For ALFF, one-sample *t*-test was performed using SPM8 in the whole frequency band in two groups to find the regions with higher-than-mean ALFF.

To investigate the main/interaction effects of/between group and frequency band in ALFF, 2 × 4 within-subject repeated-measures analysis of variance (ANOVA) was used to minimize the chance of type I error. Group (the healthy controls vs. the aCAS patients) was set as a within-subject factor and frequency band (Slow-2 vs. Slow-3 vs. Slow-4 vs. Slow-5) was served as a repeated-measures factor. 2 × 2 ANOVA was performed to deeply explore the interaction between group and frequency with the consideration of two frequency bands (Slow-5 vs. Slow- 4).

Then Two-sample *t*-test was performed to determine between-group differences in each frequency band, respectively. Significant different regions were shown on MNI templates.

Finally, brain regions with significant between-group differences were defined as regions of interest (ROIs); Spearman analysis was performed to detect the correlations between the mean ALFF value in the ROIs and cognition scores.

### Neural Network Modeling

Two types of cognition impairment analysis models were designed in this study: cognition impairment classifier and cognition scores predictor.

(1) Cognition impairment classifiers were designed using SVM model (Cristianini, 2000) in two schemes.

In SVM classifier scheme I (Supplementary Figure S1A), eight ROIs were found in the whole frequency bands. The mean ALFF value of the eight ROIs were set as the model inputs. In contrast in scheme II (Supplementary Figure S1B), 13 ROIs were found in the sub frequency bands in sum (0 in Slow-2, 6 in Slow-3, 4 in Slow-4, and 3 in Slow-5). The mean ALFF value of the 13 ROIs were set as the model inputs. In both two schemes, the group index was set as the only output of the classifier. The group with cognition impairment was marked as number -1 whereas the healthy control group was marked as number 1. LIBSVM (Chang and Lin, 2011) was used in the SVM modeling. Classification accuracy (1) and area under curve (AUC) were used to access the performance of the classifier:

$$\text{Classification accuracy=}\frac{N_{corrected}}{N_{sample}}\times 100\%$$

where *N*_corrected_ is the number of sample classified correctly and *N*_sample_ the total number of the training set and the test set. AUC is the area under the ROC of the SVM classifier.

(2) Cognition scores predictors were designed using BPNN model (Hagan, 1996) in three schemes.

Scheme I (Supplementary Figure S2A) was designed based on the significant correlation found between the cognition scores and the mean ALFF value in the ROI. The mean ALFF in the three ROIs which were significantly correlated to DR score were set as the input of the predictor; In scheme II (Supplementary Figure S2B), the mean ALFF value in the eight ROIs found in the whole frequency bands were set as the model inputs, which is the same as the inputs in the scheme I of the SVM classifier model. In scheme III (Supplementary Figure S2C), the mean ALFF value in the thirteen ROIs found in the all sub frequency bands were set as the model inputs, which is the same as the inputs in the scheme II of the SVM classifier model. The only one output in the predictor is DR score of the subject. Neural Network toolbox in Matlab (Mathworks Co., United States) was used in the BPNN modeling. Mean absolute error (MAE) was used to represent the performance of the predictor, and was calculated as follow,

$$MAE=\frac{\Sigma abs\left(y_{i}-t_{i}\right)}{N_{sample}}$$

where *y_i_* is the predicted results using trained BPNN model, *t_i_* the actual (target) DR score of the subject, and *N*_sample_ the total number of the training set and/or the test set.

In this study, there were 43 subjects, in which, the front 23 subjects in order were used as the training set and the remain 20 subjects were used as test set. The order of the data arranged as training set and test set was disrupted randomly 1000 times to test the average performance of the designed models (mean ± std).

## Results

### Subjects Characteristics and Neuropsychological Evaluation

In this study, we enrolled 19 aCAS patients and 24 healthy controls. No significant difference was found in educational years, gender, age and vascular risk-related factors (Hypertension, Diabetes mellitus, and Hypercholesterolemia). Compared with healthy controls (**Table 1**), aCAS patients showed significantly poorer performances on global cognition (represented by MMSE and MoCA), memory (represented by DST and RAVLT), and executive function (represented by DSST).

### AlFF Patterns in Whole Frequency Band (0.01–0.25 Hz)

**FIGURE 1:**
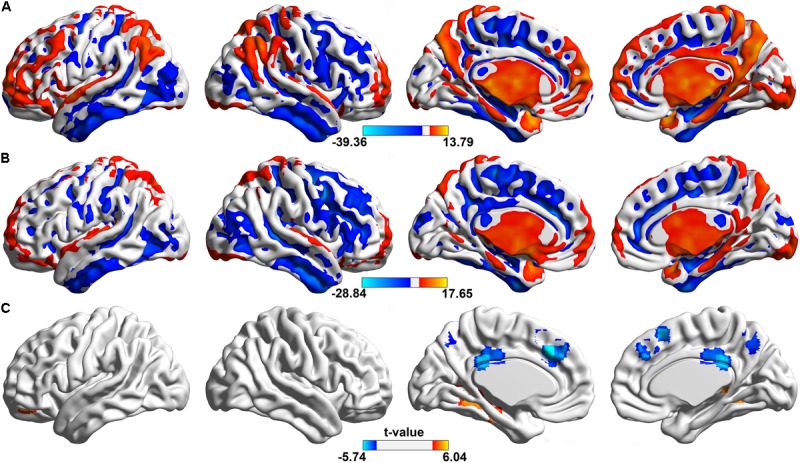
Spatial distributions of significant ALFF in the whole frequency band (0.01–0.25 Hz). The regions showing the spatial distribution of the ALFF patterns in **(A)** control group, **(B)** aCAS group (one sample *t*-test, voxel size > 100, *p* < 0.05, FDR corrected) and **(C)** their between-group difference (two sample *t*-test, voxel size > 10, *p* < 0.05, FDR corrected). In one sample *t*-test, hotter color indicated the higher ALFF than the mean ALFF of the whole brain while cooler color meant the lower ALFF. In two sample *t*-test, hot color indicates the increased ALFF in the aCAS group than that in the control group, whereas cool color means the opposite.

Firstly, we found in the whole frequency band (0.01–0.25 Hz), both two groups showed a significant higher ALFF value than that of global average in the brain including (**Figures 1A,B**): bilateral dorsomedial prefrontal (dmPFC), cingulate cortex (CC), precuneus (PCu), supplementary motor area (SMA), thalamus, parahippocampus, superior temporal gyrus and inferior parietal lobule (*p* < 0.05, FDR corrected).

Compared with healthy control (**Figure 1C** and **Table 2**), aCAS group showed significant ALFF decrease in bilateral dmPFC, CC, PCu, and SMA. At the same time, ALFF increase was observed mainly in bilateral fusiform, hippocampus, parahippocampus, right thalamus, and left orbital frontal gyrus (*p* < 0.05, FDR corrected).

**Table 2:** In whole frequency band, details of the clusters showing significant between-group differences on ALFF at the given threshold (*p* < 0.05, FDR corrected).

Brain regions	MNI coordinates			BA	L/R	Voxels	T-value
					
	*X*	*Y*	*Z*				
FFG/LING/PHG/HIP	-33	-60	0	19/36	L	158	5.7694
FFG/PHG	33	-48	-3	19	R	29	5.4096
HIP/THA	18	-33	3	–	R	56	5.5470
ORBmid	-24	48	-18	11	L	12	4.8573
SFGmed/ACC/DCC	-3	27	39	6/9/32	L&R	56	-5.7360
PCC/DCC	0	-36	27	23/31	L&R	49	-5.4628
SMA	3	21	54	8	L&R	15	-5.2828
PCu	0	-63	54	7	L&R	32	-4.7468


**FFG, fusiform gyrus; LING, lingual gyrus; PHG: parahippocampal gyrus; HIP, hippocampus; THA, thalamus; ORBmid, middle frontal gyrus, orbital part; ORBmed, superior frontal gyrus, medial part; ACC, anterior cingulate cortex; DCC, median cingulate and paracingulate gyri; PCC, posterior cingulate cortex; SMA, supplementary motor area; PCu, precuneus.**

### Main Effect of the Group and Frequency Factors

**FIGURE 2:**
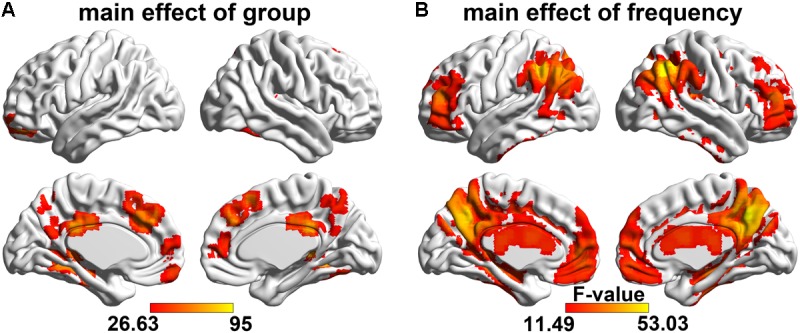
Main effect of the group and frequency factors on ALFF. The regions showing the spatial distribution of the significant **(A)** group main effect and **(B)** frequency main effect (*F*-test, voxel size > 20, *p* < 0.05, FWE corrected). The hot color represents the greater main effect of the factors on the ALFF.

The main effect of the group factor was shown in **Figure 2A** and **Table 3**. Brain regions with a main effect of group factor on ALFF mainly includes: bilateral dmPFC, CC, PCu, SMA, fusiform, hippocampus, parahippocampus, thalamus, left orbital frontal gyrus and gyrus rectus (*p* < 0.05, FWE corrected).

**Table 3:** Details of the clusters showing significant main effect of the group factor on ALFF at the given threshold (*p* < 0.05, FWE corrected).

Brain regions	MNI coordinates			BA	L/R	Voxels	*F*-value
					
	*X*	*Y*	*Z*				
FFG/HIP/PHG/THA/ITG	-33	-60	0	19	L	479	76.6334
ORBmid/ORBsup/REC	-24	51	-15	11	L	89	63.3996
FFG/HIP/PHG/THA	36	-48	-6	19	R	176	83.9097
SFGmed/ACC	3	54	3	9/10	L&R	35	42.8731
DCC/PCC	0	-36	27	23/31	L&R	81	70.1513
SFGmed/SMA/ACC/DCC	0	36	36	6/8/9/32	L&R	147	65.2123
PCu	0	-63	57	7/31	L&R	67	47.3899


**ITG, inferior temporal gyrus; ORBsup, superior frontal gyrus, orbital part; REC, rectus.**

A significant frequency (involving four sub-bands) main effect on the ALFF was observed (**Figure 2B** and **Table 4**) widely distributed in the cortical and subcortical structure, including the prefrontal gyrus, temporal gyrus, occipital gyrus, parietal gyrus, bilateral CC, PCu, hippocampus, parahippocampus, thalamus, and the basal ganglia area (*p* < 0.05, FWE corrected).

**Table 4:** Details of the clusters showing significant main effect of the frequency factor on ALFF at the given threshold (*p* < 0.05, FWE corrected).

Brain regions	MNI			BA	L/R	Voxel s	*F*-valu e
					
	*X*	*Y*	*Z*				
HIP/ITG/SFG/THA/PHG	39	-48	9	6/13/20/32/36	L&R	5370	39.1918
SFGmed/ORBsupmed/ACC/REC	-6	51	0	9/10/11/32	L&R	431	29.1149
PCu/DCC/PCC/CAL/SPG	3	-57	39	5/7/23/31	L&R	1392	52.6756
MFG/IFGtri/ORBinf/ORBmid	-39	48	15	10/11/46	L	245	32.4143
MFG/ORBinf/ORBsup/ORBmid/SFG	42	48	15	8/10/11/46	R	265	29.0655
IPL/ANG/MOG/MTG/SMG/SPG	-57	-48	42	7/19/39/40	L	762	44.3019
ANG/IPL/SMG/MOG/MTG/STG	51	-57	39	7/19/39/40	R	690	53.0286


**SFG, superior frontal gyrus; CAL, calcarine fissure and surrounding cortex; SPG, superior parietal gyrus; MFG, middle frontal gyrus; IFGtri, inferior frontal gyrus, triangular part; ORBinf, inferior frontal gyrus, orbital part; IPG, inferior parietal lobe; ANG, angular gyrus; MOG, middle occipital gyrus; MTG, middle temporal gyrus; SMG, supramarginal gyrus; STG, superior temporal gyrus*.*

Taken Slow-4 and Slow-5 as an example (Supplementary Figure S3 and Supplementary Table S1), we observed that greater ALFF in Slow-4 than Slow-5 mainly included bilateral thalamus, hippocampus, parahippocampus, caudate nucleus and insula. In contrast lower ALFF in Slow-4 than Slow-5 was found in orbital frontal cortex, dmPFC, occipital gyrus and inferior temporal gyrus.

### Interaction Between the Group and Frequency Factors

There was no significant interaction between frequency bands and two groups when four sub frequency bands were considered (*P* < 0.05, Alphasim corrected).

**FIGURE 3:**
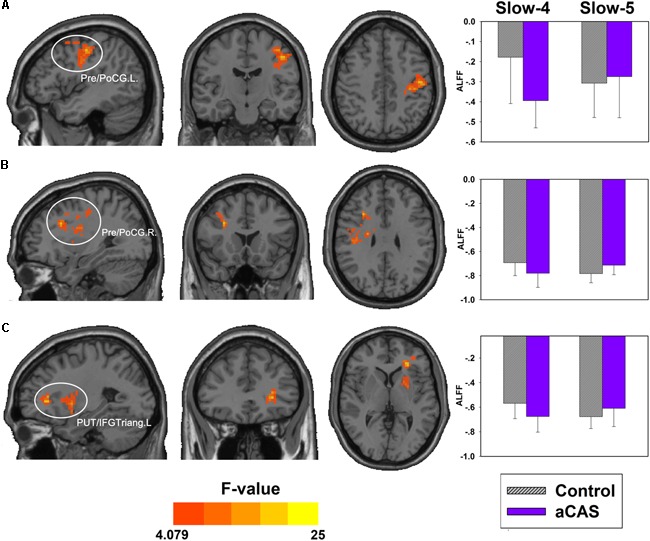
Interactions between group and frequency bands (slow-4 and slow-5). The regions showing significant interactions on ALFF (hot colors) includes: **(A)** the left precentral and postcentral cortex, **(B)** the right precentral and postcentral cortex, and **(C)** left putaman and Inferior frontal gyrus triangular part. The bar maps show the mean ALFF values in these regions.

However, the ANOVA test in ALFF when only Slow-4 and Slow-5 bands were considered showed significant interactions between group and frequency in three clusters (**Figure 3** left part and **Table 5**): left putamen/triangle part of inferior frontal gyri, left precentral/postcentral gyrus, and right precentral/postcentral gyrus (*P* < 0.05, Alphasim corrected).

**Table 5:** Brain regions showing significant interaction effects between group and frequency (slow-4 and slow-5) on ALFF.

Brain region	*x*, *y*, *z*	BA	Cluster size	*F*-value	Slow-4, *t*, *p*	Slow-5, *t*, *p*
Pre/PoCG. L	-45,-15,45	3/4/6	270	20.1441	-0.2159,<0.001	0.0327,0.5706
Pre/PoCG. R	30,18,30	3/4	362	17.5821	-0.0881,0.0143	0.0695,0.0055
PUT/IFGtri. L	-27,33,3	–	151	20.3535	-0.1069,0.0082	0.0684,0.0453


**PreCG, precentral gyrus; PoCG, postcentral gyrus; PUT, lenticular nucleus, putamen*.*

Furthermore, in left precentral/postcentral gyrus, we found greater ALFF in the control group than that in aCAS group within Slow-4 band while no significant difference within Slow-5 band; In right precentral/postcentral gyrus and left putamen/triangle part of inferior frontal gyri, we found greater ALFF in the control group than that in aCAS group within Slow-4 band but lower ALFF in the control group than that in aCAS group within Slow-5 band (**Figure 3** right part and **Table 5**).

### ALFF Changes in Sub Frequency Bands

No significant difference between the aCAS patient and healthy control was found in Slow-2 band (*P* < 0.05, FDR corrected).

**FIGURE 4:**
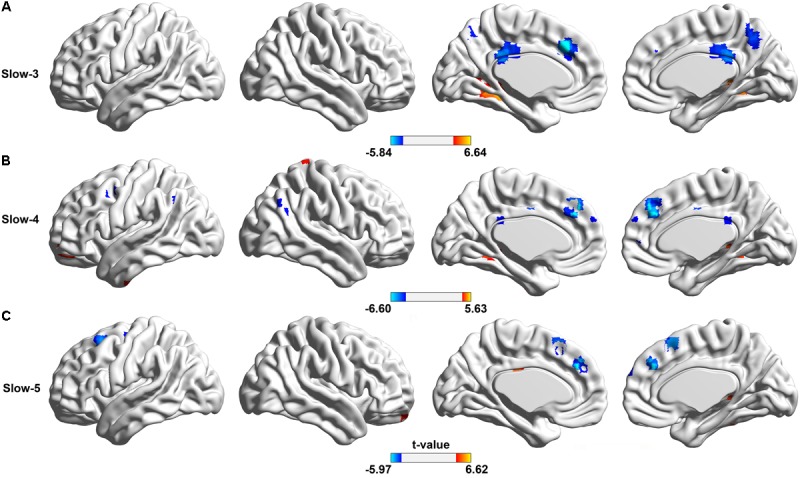
Spatial distributions of significant between group difference on ALFF of BOLD oscillations in different frequency bands. The regions showing the between-group difference in **(A)** Slow-3 band, **(B)** Slow-4 band, and **(C)** Slow-5 band, respectively, (two sample *t*-test, voxel size > 10, *p* < 0.05, FDR corrected). In two sample *t*-test, hotter color indicated the increased ALFF in the aCAS group than that in the control group, while cooler color meant the decreased ALFF.

In Slow-3 band, compared with the healthy control (**Figure 4A** and Supplementary Table S2), aCAS group showed significant ALFF decreases in bilateral CC, PCu and left dmPFC; meanwhile, significant ALFF increases were also observed in bilateral lingual, fusiform, hippocampus, parahippocampus and right thalamus (*p* < 0.05, FDR corrected).

In Slow-4 band, compared with the healthy controls (**Figure 4B** and Supplementary Table S3), aCAS group showed significant ALFF decreases in bilateral dmPFC; meanwhile, significant ALFF increases were observed in left middle frontal gyrus, orbital part and right hippocampus, thalamus, parahippocampus and fusiform (*p* < 0.05, FDR corrected).

In Slow-5 band, compared with the healthy control (**Figure 4C** and Supplementary Table S4), aCAS group showed significant ALFF decreases in bilateral dmPFC, SMA and left middle frontal gyrus (*p* < 0.05, FDR corrected).

### Correlations Between Cognitive Scores and ALFF Changes in aCAS Patients

**FIGURE 5:**
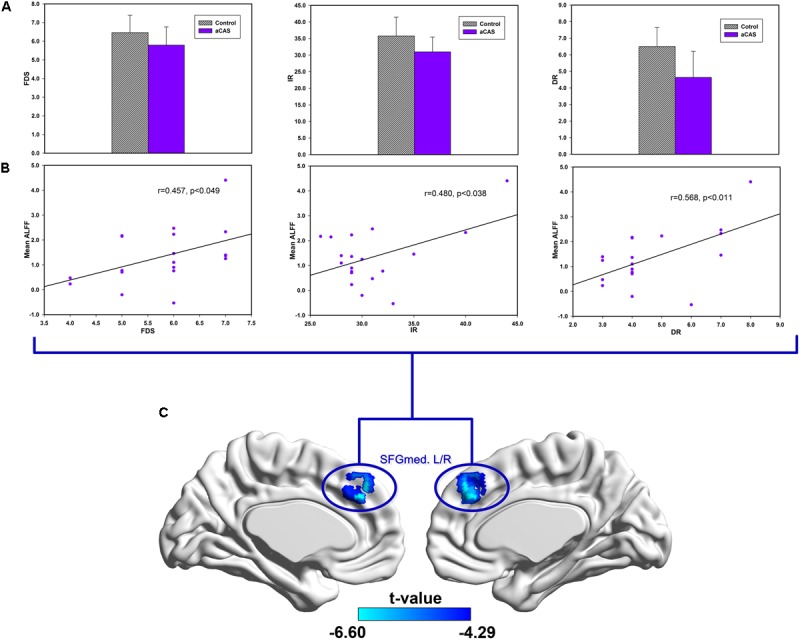
Correlations between cognitive variables and ALFF changes in the regions with significant between group difference in the Slow-3 band. **(A)** From left to the right, the bar maps show FDS, IR and DR scores between two groups. **(B)** The scatters maps and its regressions show the distribution and their significant correlation between mean ALFF in **(C)** brain regions and cognitive test score (FDS, IR, and DR), respectively.

In Slow-3 band (**Figure 5** and Supplementary Table S5), we found: (1) positive correlations between the MoCA and mean ALFF changes in the right LING/FFG/PHG cluster; (2) positive correlations between DR and mean ALFF change in the left LING/FFG/PHG/HIP cluster; (3) positive correlations between DR and mean ALFF change in the right HIP/THA cluster.

**FIGURE 6:**
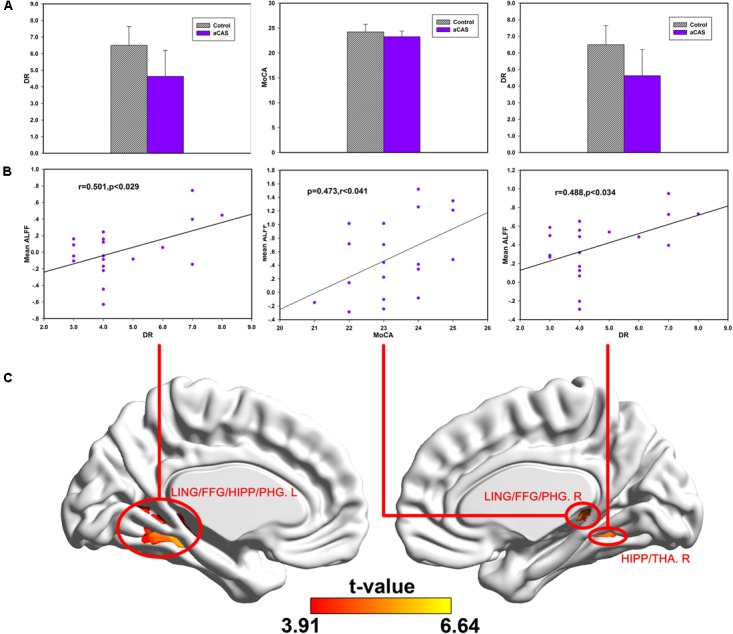
Correlations between cognitive test scores and ALFF changes in the regions with significant between group difference in Slow-4 band. **(A)** The bar maps show DR and MoCA scores between two groups. **(B)** The scatters maps and its regressions show the distribution and their significant correlation between mean ALFF in **(C)** brain regions and cognitive test score (MoCA and DR), respectively.

In Slow-4 band (**Figure 6** and Supplementary Table S6), we found positive correlations between mean ALFF change in the bilateral SFGmed cluster and FDS, IR, DR, respectively.

In Slow-5 band (Supplementary Table S7), no significant correlation was found between mean ALFF change in the clusters and cognitive scores.

### Neural Network Modeling

**FIGURE 7:**
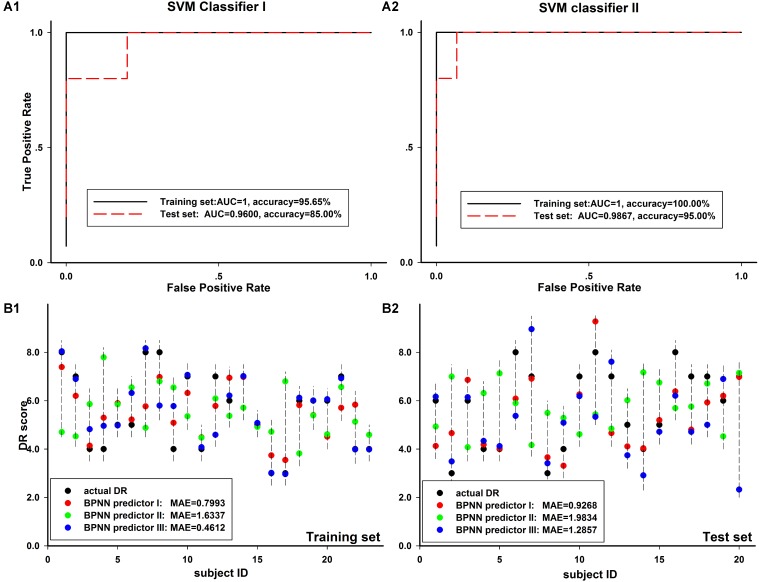
Comparison of different Neural network modeling results. **(A1,A2)** The ROC curve of training set and test set in two SVM classifier models, including 8-inputs **(A1)** and 13-inputs **(A2)** schemes, described in Section “Materials and Methods” in detail. The classification accuracy and AUC value for each scheme was shown in the corresponding figure. **(B1,B2)** The prediction results of three BPNN predictor model schemes in training set **(B1)** and test set **(B2)**. The prediction MAE for each scheme was shown in the corresponding figure.

As seen in **Table 6**, in the two schemes of SVM classifier model, we got an accuracy of above 90% and the AUC value of above 0.99 on both training set and test set. The performance of Scheme I was significantly better than that of Scheme II for both the training and the test set, *p* = 0.023/*p* = 0.041 in training set and *p* < 0.001/*p* < 0.001 in test set for classification accuracy/AUC value. In **Figures 7A1,A2**, we showed a typical example of SVM classifier models. In the example, we also found better performance in scheme I than that in scheme II, whether on the training set or the test set.

**Table 6:** The averaged performance of the SVM classifiers.

Classifier accuracy/AUC	Training set	Test set
SVM classifier I	96.20% ± 2.72%/0.9984 ± 0.0036	94.27% ± 4.93%/0.9920 ± 0.0118
SVM classifier II	97.87% ± 2.17%/0.9999 ± 0.0006	97.51% ± 2.76%/0.9973 ± 0.0066


As seen in **Table 7**, in the three schemes of BPNN predictor model, we got an average MAE of about 1.0–1.2 on training set and about 1.7–2.0 on test set. We found the average MAE of schemes I and III was close and scheme II was the worst. In **Figures 7B1,B2**, we showed a typical example of BPNN predictor models and found the similar results as shown in **Table 7**.

**Table 7:** The averaged performance of the BPNN predictors.

Predictor MSE	Training set	Test set
BPNN predictor I	1.0334 ± 0.2246	1.4992 ± 0.3675
BPNN predictor II	1.2824 ± 0.6600	2.0027 ± 0.5235
BPNN predictor III	1.1806 ± 0.2133	1.9240 ± 0.7480


## Discussion

As we know, this is the first work to explore the frequency-dependent ALFF changes from resting-state BOLD signals in aCAS patients. In this study, we analyzed ALFF changes in the aCAS patients within four sub frequency bands (Slow-5, Slow-4, Slow-3, and Slow-2 bands). A set of brain regions exhibited significant differences in ALFF were found between two groups and among the sub frequency bands.

Unfortunately, we found no significant interaction between the group and frequency bands when four sub frequency bands were considered. But we found that several brain regions (left putamen, triangular part of inferior frontal gyrus and bilateral precentral/postcentral gyrus) showed significant interaction when only Slow-4 and Slow-5 bands were considered. In these brain regions, significantly greater ALFF in healthy control than that in aCAS patients was found within Slow-4 band but significantly lower ALFF in healthy control than that in aCAS patients was presented within Slow-5 band, implied that aCAS patients had abnormal ALFF in local brain neural activity and these abnormalities were frequency-dependent, especially across Slow-4 and Slow-5.

### Differences in ALFF Between Frequency Bands

From the frequency main effects, we found that the significant ALFF changes varied in different frequency bands in many cortex regions (frontal gyrus, occipital gyrus, temporal gyrus, and parietal gyrus), hippocampus structures and the subcortical structures, including thalamus and basal ganglia.

Neural oscillation in lower frequency mainly occur on the large cortex structure and exhibits higher power while higher frequency neural activities are mainly found in subcortical structures and has lower power (Baria et al., 2011; Zhang et al., 2013). Taken Slow-4 and Slow-5 as an example, we observed that greater ALFF in higher frequency band (Slow-4) than that in lower frequency band (Slow-5) mainly presented in subcortical regions, including thalamus and caudate nucleus. In contrast greater ALFF in lower frequency band (Slow-5) than higher frequency band (Slow-4) was found in cortical regions, including orbital frontal, dorsomedial prefrontal, occipital and temporal cortex. These were consistent with the previous frequency-dependent RfMRI studies (Zuo et al., 2010; Han et al., 2011; Yu et al., 2014) and an animal experiments (Pan et al., 2013). Neural connections are mainly localized and neural oscillation period is limited to the size of neural pool involved in a given period (Csicsvari et al., 2003; Buzsaki and Draguhn, 2004). Lower frequency neural activities were related to the integration of large-scale neural networks and long-distance neural connection (Csicsvari et al., 2003). It may be mediated by cortex areas, especially the key nodes of the network (Salvador et al., 2005; Tomasi and Volkow, 2010). In contrast, signals from higher frequency band have been associated with local neural activities and short-distance connectivity (Buzsaki and Draguhn, 2004; Salvador et al., 2005), which may be found in the more primitive subcortical regions (Buzsaki and Draguhn, 2004). However, we found higher ALFF in hippocampus and medial temporal lobe (parahippocampal and fusiform gyrus) in Slow-4 than that in Slow-5, which was inconsistent with previous statements. Consider hippocampus, parahippocampal gyrus, fusiform gyrus and thalamus are adjacent in anatomical structures, the higher frequency local oscillation can easily implement the synchronization and integration of information in this local structure (Buzsaki and Draguhn, 2004).

### Differences in ALFF Between aCAS Patient and Healthy conBrain

In this study, Brain regions with significant between-group difference can be assign into two part: (1) cortical midline structure, including dmPFC, CC, and PCu; and (2) hippocampus and its adjacent structures, including hippocampus, thalamus, and medial temporal lobe (parahippocampal and fusiform gyrus). The significant decrease of cortical midline structure’s ALFF value illustrated its neural activities significantly declined, which indicated the possible generation mechanism of cognitive dysfunction, while the significant increase of hippocampus and its adjacent structures’ ALFF value illustrated its neural activities significantly raised, which implied the possible compensation mechanism of cognitive dysfunction.

Previous study (Rogers et al., 1977) found that compared with other-related processing, self-related processing can lead to memory enhancement and consolidation that contributes to the formation of long-term memory. The phenomenon is called “self-reference effect” and its process is called “self-reference process.” Cortical midline structure refers to the brain region located in the midline of the human cerebral cortex, including the medial prefrontal cortex, CC, and PCu. It plays an important role in self-related processing and is the basis for abstract, evaluative, and integrated self and other information processing (Uddin et al., 2007). various types of self-reference processing is closely related to the activation of the cortical midline structure. Apart from memory, it also involves important cognitive functions such as language, space, emotion, face, movement, and society (Northoff et al., 2006).

In hippocampus and its adjacent structures, hippocampus is the center of processing and integration of episodic memory in the brain (Scoville and Milner, 2000); thalamus is not only a relay hub for most afferent impulses to the cerebral cortex (including the hippocampus), but also the important information integration and coordination center (Gazzaniga, 2014); medial temporal lobe is associated with the short-term memory. In medial temporal lobe, parahippocampal gyrus is a gray matter layer around the hippocampus, which plays an important role in the encoding and extraction of memory (Megevand et al., 2014). Additionally, olfactory region in parahippocampal gyrus is the gate linking to the hippocampus with rich incoming connections; fusiform gyrus is linked to the function including color information processing and the recognition of face, body, and word (Uono et al., 2017).

Based on the analysis above, we speculate that cognitive dysfunction in aCAS patients may be related to the decline of neural activity in the cortical midline structure, especially for long-term memory. At the same time, possible compensatory mechanisms are formed: in hippocampus and its adjacent structures, short-term memory (medial temporal lobe) and memory processing (hippocampus) related neural activity significantly enhanced and was used to compensate for the decline of long-term memory-related cognitive functions.

Based on the “binding-by-gamma” hypothesis (Engel et al., 2001), as long as the frequency of the coupled neural oscillator remain similar, oscillation synchrony can be sustained even with very weak synaptic links. This synchrony allows activated neural oscillators link with each other temporally and form the corresponding function. Among them, low-frequency neural activity is associated with the integration of large-scale neural networks and long-distance neural connections, whereas high-frequency neural activity is associated with local neural networks and short-range neural connections.

In cortical midline structure, dmPFC, CC, and PCu are not adjacent with each other in anatomy. Low-frequency neural oscillators between them allow them to form long-distance temporary network connections and the corresponding functions. The decline of their low-frequency neural activity may affect these temporary connections and functions. Such as in **Figure 6**, the ALFF value of dmPFC in lower frequency band is significant positive Related to FDS, IR, and DR, which are linked to the cognition function of language learning, attention and memory. Similarly, the possible local compensatory behavior can also be enhanced by increasing the neural activity in hippocampus and its adjacent structures in higher frequency band. As shown in **Figure 5**, the ALFF value of the hippocampus and its adjacent structure in higher frequency band is significantly positively correlated with DR and MoCA which are linked to the cognition function of global cognition, memory and attention.

Oscillations with different frequency might carry different dimensions of brain integrations. Low frequency oscillation synchronized the large-scale network space and could bind together specific assembles by the proper higher frequency local oscillations (Engel et al., 2001; Varela et al., 2001; Sirota et al., 2003; Steriade and Timofeev, 2003). However, the underlying mechanism of neural activities with different frequency oscillations and their advanced brain functions remain unclear and need to be explored in the future.

### Classification Model and Prediction Model

Both two SVM classifier schemes and three BPNN predictor schemes achieved good diagnosis results. It meant BOLD signal in significantly altered brain regions in aCAS patients are valuable in the analysis of aCAS. The segmentation of physiological sub-bands and exaction of information within these sub-bands provides more information, for example, the neural oscillation difference in large scale neural network process and local neural process, separately. These are useful and helpful in the recognition of the cognition impairment of aCAS patients.

In sum through the modeling, we found the mean ALFF in the regions with significant between-group difference on ALFF in different frequency bands could be used as neuroimaging markers for the cognition impairment assessment of aCAS patients. The modeling results in classification and prediction can effectively verify it. The built models could be a useful tool in the clinical diagnosis and the pathophysiology mechanism exploration of cognitive impairment for aCAS patients.

### Limitation

It should be noted there are several limitations in this study. First, the sample size was relatively small, larger sample size can make the built model more reliable and robust. aCAS patients with different handedness and different side of stenosis could exhibit different cognition impairment. More patients with different handedness and different side of stenosis should be recruited in the future work. Secondly, the effects of respiratory and heart rhythm could not be eliminate completely with a relatively low sampling rate (TR = 2 s), especially in the analysis within relatively high frequency band (e.g., Slow-2 band). Thirdly, to explore the mechanism of CAS leading to cognitive dysfunction, the relationship between local neural activities characteristics and corresponding cerebral perfusion should be discussed in the future, ASL method may be a good candidate. Finally, the extent of Carotid artery stenosis and different treatments (carotid artery stenting and carotid endarterectomy) (Takaiwa et al., 2009; Feliziani et al., 2010; Wasser et al., 2012; Germano et al., 2014) should be considered to confirm their effects to the cognition impairment in the future.

Additionally, ALFF method can be used more in-depth in many clinical situations, for example, to construct the brain structural and functional network, the analysis of the network connectivity changes may provide an in-depth insight into the physiological and pathological mechanism of interested brain issues. These will be considered in our future work.

## Conclusion

In this study we explored the frequency-dependent characteristics of the abnormalities of brain function in aCAS patients. First, we examine the spatial patterns of the ALFF change in aCAS patients within four different frequency bands; then we analyze the correlation between these abnormal changes and the cognition level of aCAS patient; finally, the cognition impairment diagnosis model and prediction model were built using these frequency-dependent characteristics of LFO abnormalities. In all, the frequency-dependent abnormalities of LFO in aCAS not only reveal the pathophysiology of cognitive impairment of aCAS, but also can be used as neuroimaging marker in the diagnosis and assessment of cognition impairment for the aCAS patients.

## Author Contributions

FX processed the fMRI images, analyzed the data, and drafted the manuscript. TW recruited the aCAS patients and performed the neuropsychological test for the aCAS patients. HX and JZ designed this work and revised the manuscript. LG analyzed the data and revised the manuscript. JF and ZS collected the RfMRI data of the subjects in the MR scanning.

## Conflict of InterestStatement

The authors declare that the research was conducted in the absence of any commercial or financial relationships that could be construed as a potential conflict of interest.

## Footnotes

1 http://rfmri.org/dpabi
