# Supplementary material for: Frequency-Dependent Changes of the Resting BOLD Signals Predicts Cognitive Deficits in Asymptomatic Carotid Artery Stenosis
Source: Front Neurosci. 2018 Jun 21;12:416. doi: 10.3389/fnins.2018.00416 (PMC6021536; doi:10.3389/fnins.2018.00416)
Supplement: Supplementary file 1 [file Data_Sheet_1.DOC]

**Frequency-dependent changes in the amplitude of low frequency fluctuations predicts the cognition impairment in "asymptomatic" carotid artery stenosis**

Feng Xiao^1#^, Tao Wang^2,3#^, Lei Gao^1^, Jian Fang^1^, Zhenmeng Sun^1^, Haibo Xu^1*^, Junjian Zhang^2*^

^1^ Department of Radiology, Zhongnan Hospital of Wuhan University, Wuhan, China

^2^ Department of Neurology, Zhongnan Hospital of Wuhan University, Wuhan, China

^3^Department of Neurology, The First College of Clinical Medical Science, China Three Gorge University, Yichang, China

^#^These authors contribute equally to this work

^*^correspondence:

Haibo Xu: xuhaibo1120@hotmail.com

Junjian Zhang: wdsjkx@163.com

**Supplemental Material**

**Figure Legends**

**Figure S1. Two schemes of SVM classifier models.** (A) 8-inputs model and (B)13-inputs model, described in Methods in detail.

**Figure S2. Three schemes of BPNN predictor models.** (A) 3-inputs model, (B)8-inputs model and (C) 13-inputs model, described in Methods in detail.

**Figure S3. Spatial distributions of significant difference on ALFF of BOLD oscillations between Slow-5 and Slow-4 band.** In two sample t-test, hotter color indicated the greater ALFF in Slow-5 band than that in Slow-4 band, while cooler color meant the lower ALFF. (two sample t-test, voxel size>10, p<0.05, FDR corrected)

**Figures**

**Figure S1**

**
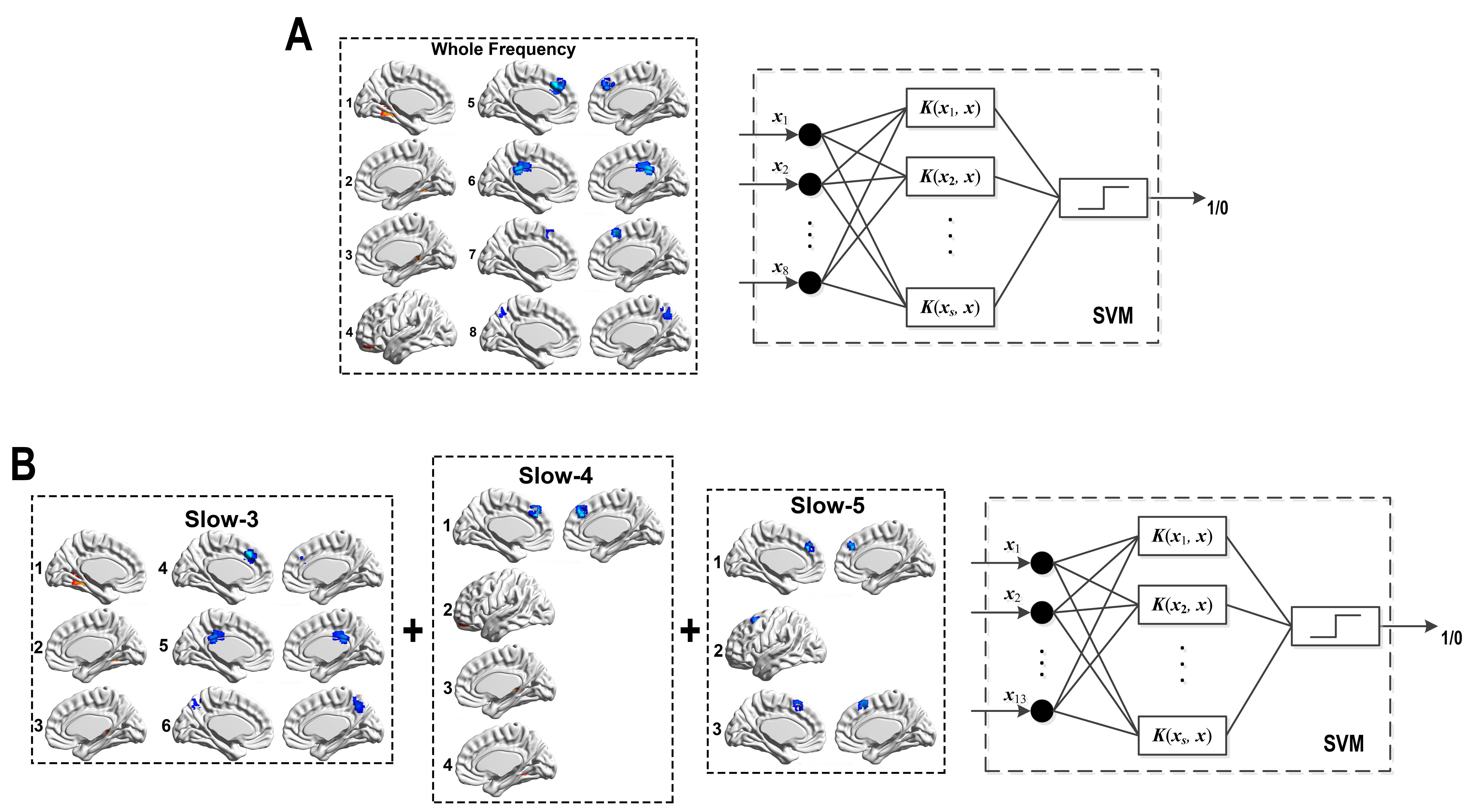
**

**Figure S2**

**
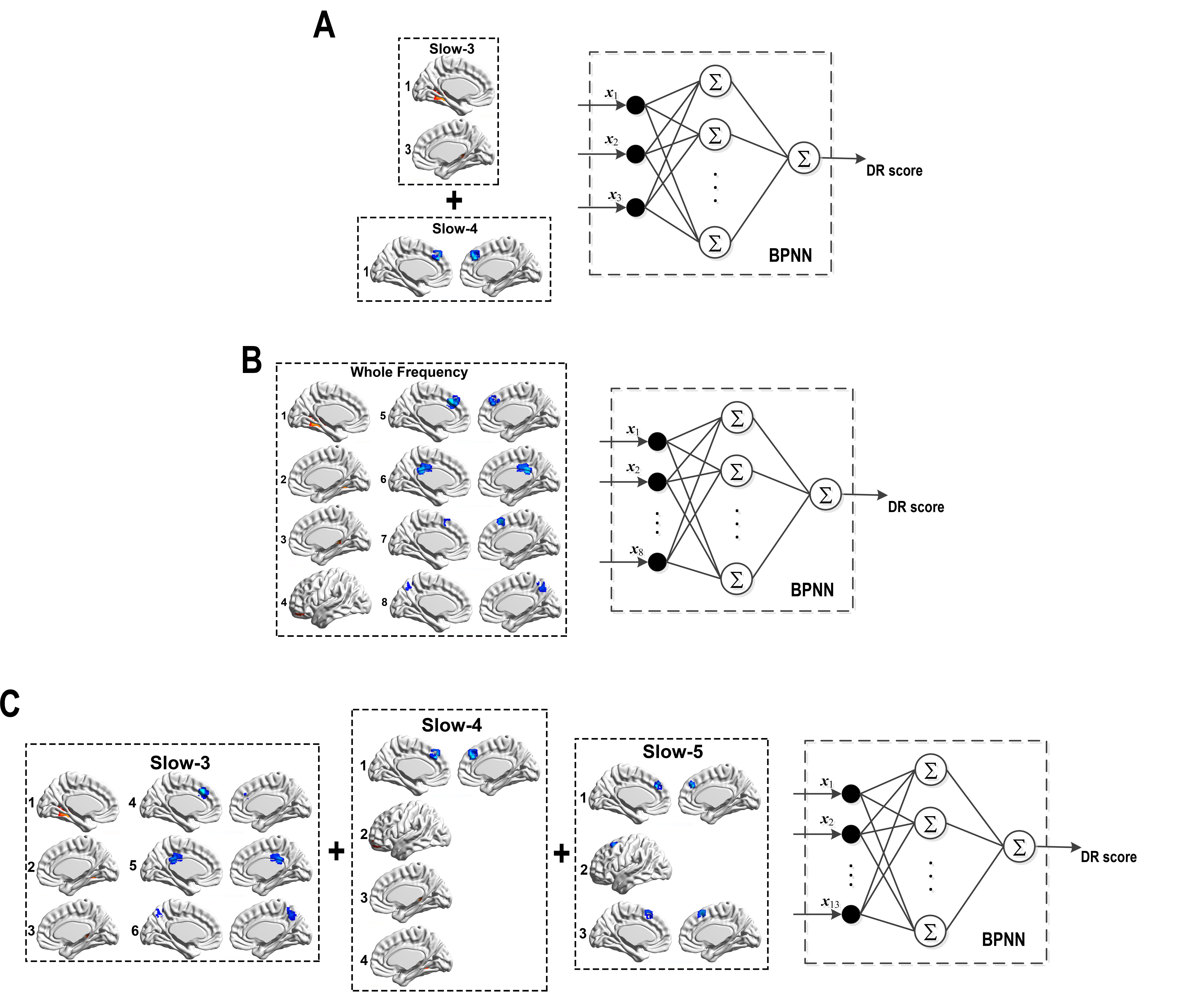
**

**Figure S3**

**
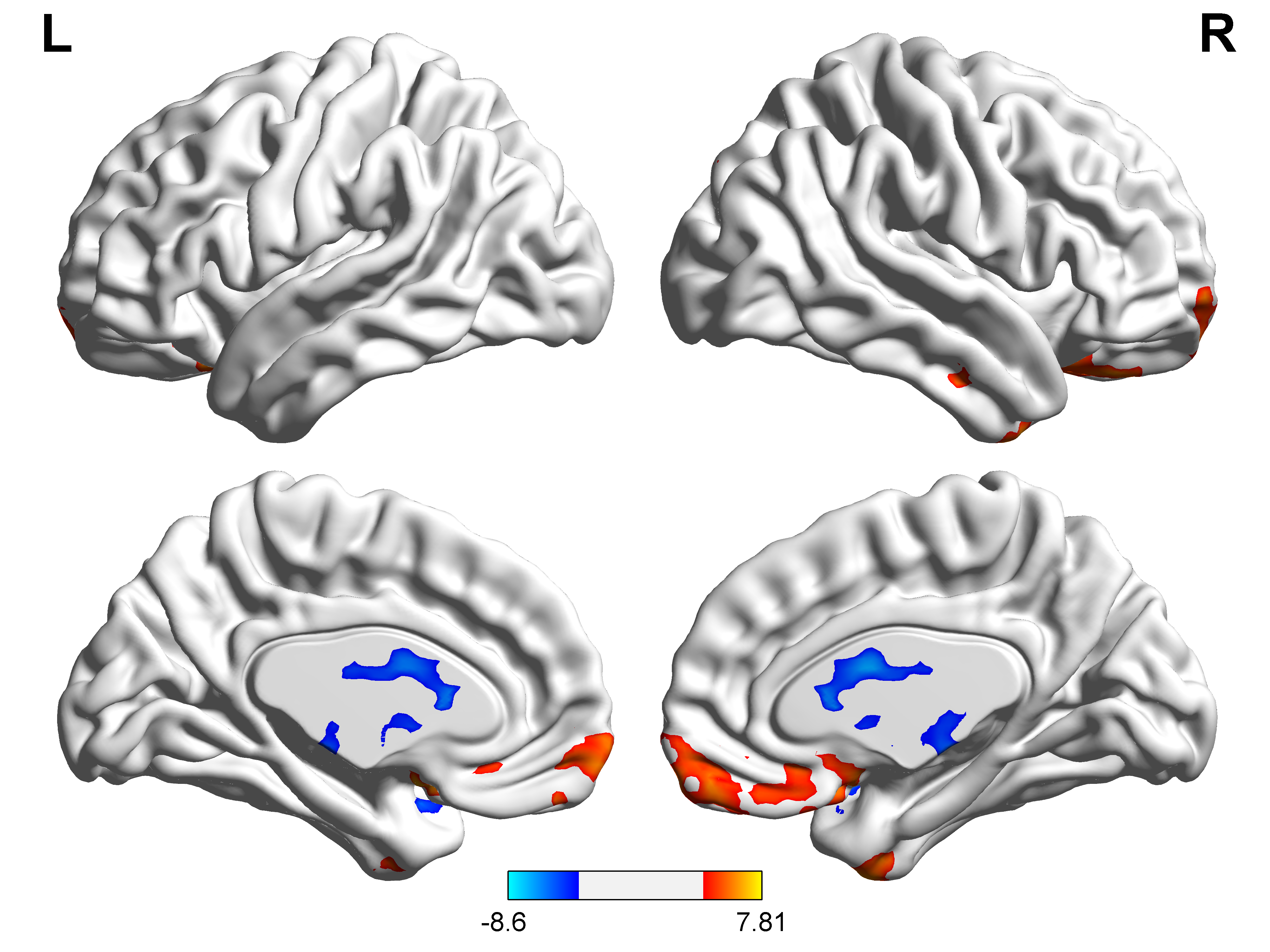
**

**Tables**

**Table. S1 Detailed information for clusters showing significant frequency ALFF differences at the given threshold between Slow-4 band and Slow-5 band. (p < 0.05, FDR corrected).**

| Brain regions | MNI coordinates | | | BA | L/R | voxels | T-value |
| --- | --- | --- | --- | --- | --- | --- | --- |
|  | X | Y | Z |  |  |  |  |
| **FFG/ ITG/TPOmid** | 30 | 12 | -45 | 20/38 | R | 106 | 7.2321 |
| **FFG/ ITG/TPOmid** | -30 | 3 | -45 | 20/38 | L | 26 | 6.4542 |
| **ITG/MTG** | 54 | -12 | -30 | 20 | R | 34 | 6.4124 |
| **ORBsup/ORBmed/ORBinf/ORBmid** | -15 | 9 | -18 | 10/11/25/47 | L/R | 1210 | 7.8137 |
| **SOG/MOG** | 27 | -87 | 39 | 19 | R | 20 | 5.0502 |
| **CAU/THA/HIP/PHG** | -3 | -21 | -60 | - | L/R | 2108 | -8.6035 |
| **TPOsup** | -33 | 9 | 24 | 38 | L | 117 | -6.3493 |
| **TPOsup** | 45 | 9 | -18 | 38 | R | 44 | -6.5005 |
| **INS** | 45 | 0 | -3 | - | R | 33 | -6.2971 |

**Table. S2. Detailed information for clusters showing significant group ALFF differences at the given threshold in Slow-3 band. (p < 0.05, FDR corrected).**

| Brain regions | MNI coordinates | | | BA | L/R | voxels | T-value |
| --- | --- | --- | --- | --- | --- | --- | --- |
|  | X | Y | Z |  |  |  |  |
| **LING/FFG/PHG/HIP** | -36 | -42 | -9 | 19 | L | 118 | 5.5701 |
| **LING/FFG/ PHG** | 33 | -48 | -3 | - | R | 32 | 5.4753 |
| **HIP/THA** | 18 | -31 | 3 | - | R | 42 | 5.7527 |
| **SFGmed/ACC/DCC** | -3 | 27 | 39 | 6/9/32 | L | 28 | -5.8353 |
| **PCC/DCC** | 0 | -36 | 27 | 23 | L/R | 28 | -5.0040 |
| **PCu** | 0 | -63 | 54 | 7 | L/R | 36 | -4.7067 |

**Table. S3. Detailed information for clusters showing significant group ALFF differences at the given threshold in Slow-4 band. (p < 0.05, FDR corrected).**

| Brain regions | MNI coordinates | | | BA | L/R | voxels | T-value |
| --- | --- | --- | --- | --- | --- | --- | --- |
|  | X | Y | Z |  |  |  |  |
| **SFGmed** | 0 | 36 | 36 | 6/8/9 | L/R | 31 | -6.6039 |
| **ORBmid** | -21 | 57 | -9 | 11 | L | 13 | 4.6212 |
| **HIP/THA** | 21 | -33 | 6 | - | R | 10 | 4.8183 |
| **FFG/PHG** | 36 | -48 | -6 | - | R | 12 | 4.9521 |

**Table. S4. Detailed information for clusters showing significant group ALFF differences at the given threshold in Slow-5 band. (p < 0.05, FDR corrected).**

| Brain regions | MNI coordinates | | | BA | L/R | voxels | T-value |
| --- | --- | --- | --- | --- | --- | --- | --- |
|  | X | Y | Z |  |  |  |  |
| **SFGmed** | 0 | 42 | 33 | 9 | L/R | 17 | -5.9667 |
| **MFG** | -27 | 15 | 54 | 8 | L | 12 | -5.2037 |
| **SMA** | 3 | 21 | 54 | 8 | L/R | 15 | -5.308 |

**Table. S5. Pearson correlation between cognitive test scores and ALFF changes in the regions with significant between group difference in Slow-3 band.**

| Brain regions | cognitive test | | | | | | |
| --- | --- | --- | --- | --- | --- | --- | --- |
|  | MMSE | MoCA | FDS | BDS | IR | DR | DST |
| **LING/FFG/PHG/HIP** | 0.16/0.51 | 0.10/0.67 | 0.13/0.61 | 0.16/0.51 | 0.41/0.08 | 0.50/0.03 | -0.00/0.98 |
| **LING/FFG/ PHG** | 0.14/0.57 | 0.47/0.04 | 0.24/0.32 | -0.07/0.76 | 0. 20/0.42 | 0.33/0.17 | -0.14/0.56 |
| **HIP/THA** | 0.11/0.66 | 0.20/0.42 | 0.23/0.34 | 0.14/0.57 | 0.36/0.14 | 0.49/0.03 | -0.17/0.49 |
| **SFGmed/ACC/DCC** | 0.04/0.87 | -0.34/0.15 | -0.15/0.53 | -0.06/0.82 | 0.20/0.41 | 0.05/0.84 | -0.18/0.47 |
| **PCC/DCC** | 0.15/0.54 | -0.05/0.84 | -0.04/0.86 | -0.10/0.68 | 0.15/0.55 | -0.11/0.64 | 0.15/0.54 |
| **PCu** | 0.36/0.13 | -0.14/0.57 | 0.18/0.46 | 0.08/0.74 | -0.24/0.32 | -0.24/0.32 | 0.15/0.53 |

**Table. S6. Pearson correlation between cognitive test scores and ALFF changes in the regions with significant between group difference in Slow-4 band.**

| **Brain regions** | **cognitive test** | | | | | | |
| --- | --- | --- | --- | --- | --- | --- | --- |
|  | **MMSE** | **MoCA** | **FDS** | **BDS** | **IR** | **DR** | **DST** |
| **SFGmed** | -0.22/0.38 | 0.24/0.32 | 0.46/0.05 | -0.18/0.46 | 0.48/0.04 | 0.57/0.01 | -0.02/0.95 |
| **ORBmid.L** | -0.11/0.66 | -0.18/0.46 | 0.03/0.91 | -0.17/0.50 | -0.10/0.68 | 0.04/0.87 | -0.02/0.92 |
| **HIP/THA.R** | -0.04/0.86 | 0.21/0.40 | 0.03/0.90 | 0.40/0.10 | 0.24/0.32 | 0.29/0.23 | -0.10/0.69 |
| **FFG/PHG.R** | -0.07/0.78 | 0.37/0.12 | 0.17/0.49 | -0.11/0.66 | 0.20/0.41 | 0.43/0.07 | -0.08/0.74 |

**Table. S7. Pearson correlation between cognitive test scores and ALFF changes in the regions with significant between group difference in Slow-5 band.**

| **Brain regions** | **cognitive test** | | | | | | |
| --- | --- | --- | --- | --- | --- | --- | --- |
|  | **MMSE** | **MoCA** | **FDS** | **BDS** | **IR** | **DR** | **DST** |
| **SFGmed** | -0.18/0.47 | -0.40/0.09 | -0.17/0.49 | -0.30/0.22 | 0.12/0.64 | 0.03/0.91 | 0.31/0.20 |
| **MFG.L** | -0.27/0.27 | -0.27/0.27 | 0.04/0.87 | -0.02/0.93 | 0.10/0.67 | 0.05/0.84 | 0.05/0.84 |
| **SMA** | -0.27/0.27 | 0.25/0.30 | -0.09/0.71 | -0.27/0.27 | 0.16/0.52 | -0.08/0.76 | -0.09/0.70 |
